# TQCPat: Tree Quantum Circuit Pattern-based Feature Engineering Model for Automated Arrhythmia Detection using PPG Signals

**DOI:** 10.1007/s10916-025-02169-0

**Published:** 2025-03-24

**Authors:** Mehmet Ali Gelen, Turker Tuncer, Mehmet Baygin, Sengul Dogan, Prabal Datta Barua, Ru-San Tan, U. R. Acharya

**Affiliations:** 1 Department of Cardiology, Elazig Fethi Sekin City Hospital, Elazig, Turkey; 2 Department of Digital Forensics Engineering, College of Technology, Firat University, Elazig, Turkey; 3 Department of Computer Engineering, College of Engineering, Erzurum Technical University, Erzurum, Turkey; 4 School of Mathematics, Physics and Computing, University of Southern Queensland, Toowoomba, Australia; 5 National Heart Centre Singapore, Singapore, 169609 Singapore; 6 Duke-NUS Medical School, Singapore, 169857 Singapore; 7 School of Mathematics, Physics and Computing, University of Southern Queensland, Springfield, Australia; 8 Centre for Health Research, University of Southern Queensland, Toowoomba, Australia

**Keywords:** TQCPat, Multiple feature selection, PPG signals, Arrhythmia classification, Self-organized feature engineering, Biomedical signal analyses

## Abstract

**Background and Purpose:**

Arrhythmia, which presents with irregular and/or fast/slow heartbeats, is associated with morbidity and mortality risks. Photoplethysmography (PPG) provides information on volume changes of blood flow and can be used to diagnose arrhythmia. In this work, we have proposed a novel, accurate, self-organized feature engineering model for arrhythmia detection using simple, cost-effective PPG signals.

**Method:**

We have drawn inspiration from quantum circuits and employed a quantum-inspired feature extraction function /named the Tree Quantum Circuit Pattern (TQCPat). The proposed system consists of four main stages: (i) multilevel feature extraction using discrete wavelet transform (MDWT) and TQCPat, (ii) feature selection using Chi-squared (Chi2) and neighborhood component analysis (NCA), (iii) classification using k-nearest neighbors (kNN) and support vector machine (SVM) and (iv) information fusion.

**Results:**

Our proposed TQCPat-based feature engineering model has yielded a classification accuracy of 91.30% using 46,827 PPG signals in classifying six classes with ten-fold cross-validation.

**Conclusion:**

Our results show that the proposed TQCPat-based model is accurate for arrhythmia classification using PPG signals and can be tested with a large database and more arrhythmia classes.

## Introduction

### Background

Photoplethysmography (PPG)―an electro-optical technique that measures the amount of light transmitted to, or reflected unto, a photodiode while the exposed skin ((such as the fingertip) is being illuminated with an external light source [1]―provides information on volume changes of blood flowing in the superficial issues just beneath the skin surface [2–4]. While ECG and PPG capture different physiological signals, they are intrinsically related. ECG records the heart's electrical activity, providing detailed morphological waveforms of each cardiac cycle, while PPG measures blood volume changes in peripheral circulation, which are influenced by cardiac contractions [5]. The relationship between these two signals is evident in the pulse transit time (PTT), which represents the delay between the R-peak of the ECG waveform and the arrival of the corresponding pulse wave in the PPG signal [6]. A shorter PTT typically correlates with increased arterial stiffness and higher blood pressure, whereas a longer PTT suggests lower vascular resistance [7]. Given this strong physiological correlation, PPG signals can serve as a viable alternative for arrhythmia detection, especially in wearable and non-invasive monitoring systems [8]. While ECG provides more detailed morphological information, PPG offers practical advantages such as ease of acquisition, lower cost, and suitability for continuous monitoring. With portable hardware capable of providing continuous readouts that reflect pulsatile blood flow volume, PPG offers a feasible non-invasive approach for remote ambulatory heart rate and rhythm monitoring [9, 10]. Normally, the heart beats in a predominantly regular manner. Arrhythmia is any deviation from this typical regular pulse wave pattern [11] the heartbeat becomes irregular and/or inordinately fast or slow [12], which can be directly observed on the PPG readout [13–15]. A common clinical problem, arrhythmia can be asymptomatic or manifest with symptoms like palpitations, shortness of breath, chest pressure, fatigue, and weakness [16]. Often a consequence of diverse cardiovascular disorders, arrhythmia per se can also result in heart complications like heart failure, stroke and cardiac arrest [17, 18], underscoring the need for early detection [19]. Despite lacking the ability of electrocardiography (ECG) for morphological characterization of individual heartbeats [20], PPG holds promise as a pragmatic and low-cost adjunctive screening tool for arrhythmia detection and monitoring, especially in asymptomatic and/or paroxysmal arrhythmia presentations [21]. However, ECG-based arrhythmia detection is often challenged by various signal artifacts, which can reduce classification accuracy and complicate real-time monitoring [22]. Common ECG artifacts include baseline wander (caused by respiration and electrode movement), power line interference (from electrical sources), motion artifacts (due to patient movement), and electromyographic (EMG) noise (originating from muscle contractions) [23]. These artifacts necessitate complex preprocessing techniques to ensure reliable feature extraction. In contrast, PPG signals, while not free from motion artifacts, are less affected by baseline drift and muscle noise, making them a viable alternative for continuous, wearable heart monitoring applications [24]. Given these advantages, we propose a novel PPG-based machine learning model that addresses signal noise using multilevel wavelet transformation and dynamic feature extraction.

In this work, we have developed a novel tree quantum circuit pattern (TQCPat)-based feature engineering model for automated arrhythmia detection using PPG signals. The model was trained and tested on a PPG dataset comprising sinus rhythm (normal) and five distinct arrhythmia types: premature ventricular contraction, premature atrial contraction, ventricular tachycardia, supraventricular tachycardia, and atrial fibrillation (AF).

### Literature Review

Some studies on arrhythmia detection in the literature are given as follows. Wu et al. [25] proposed Res-BiANet, a hybrid deep learning model, for detecting arrhythmias using PPG signals from 91 patients, totaling 46,827 signal segments. By combining ResNet and BiLSTM for spatial and temporal feature extraction, their model achieved an F1 score of 86.88% and an accuracy of 92.38%. Neha et al. [26] presented a method for arrhythmia detection using PPG signals. They used the MIMIC II dataset, which contained 13 PPG samples for training and 2 for testing, each sampled at 125 Hz over a 24-s period. Their results showed that the support vector machine (SVM) classifier achieved an accuracy of 97.67% with normal pulse and abnormal pulse. Paradkar et al. [27] proposed an approach for detecting cardiac arrhythmias using PPG signals, leveraging the PhysioNet Challenge 2015 dataset, which included 628 PPG recordings from bedside monitors. Their used dataset contained five types of arrhythmias: tachycardia, bradycardia, asystole, ventricular tachycardia, and ventricular fibrillation. Their approach achieved a 93% true positive rate and a 53.78% true negative rate by applying pulse quality indexing and Gaussian curve fitting to improve heart rate estimation. Neha et al. [28] used dynamic time warping (DTW) with PPG signals from the PhysioNet MIMIC-II database to detect arrhythmias. Their model achieved 95.97% accuracy, with 97% sensitivity and specificity, classifying normal, PVC, atrial flutter, and tachycardia signals. Han et al. [29] proposed a peak detection algorithm for smartwatch PPG signals to improve heart rate estimation across various arrhythmias. Using data from 16 participants, the method reduced beat-to-beat RMSE by over 40% and significantly lowered undetected beats. Liu et al. [30] developed a deep CNN model for multiclass arrhythmia detection using PPG signals. They used data from 228 patients, totaling 118,217 10-s PPG segments, to classify six types of rhythms, including sinus rhythm, premature ventricular contraction, premature atrial contraction, ventricular tachycardia, supraventricular tachycardia, atrial fibrillation. Their model achieved an overall accuracy of 85%, with a sensitivity of 75.8% and a specificity of 96.9%, demonstrating the feasibility of detecting multiple arrhythmia types through PPG signals.

### Motivation and our Model

To overcome the inherent limitations of manual screening in medical diagnostics, artificial intelligence (AI) has been increasingly utilized for various classification tasks, offering enhanced efficiency and accuracy in automated decision-making processes [31–33]. Among AI methodologies, deep learning techniques have demonstrated remarkable classification performance, particularly in biomedical applications. However, these methods often require large amounts of labeled data, extensive computational resources, and suffer from a lack of interpretability, making them less practical for real-time and resource-constrained healthcare environments [34]. Consequently, there is a pressing need for more lightweight yet highly accurate machine learning-based diagnostic models that can bridge the gap between performance and computational efficiency [35]. These models not only contribute to real-world medical applications but also support the progression toward Artificial General Intelligence (AGI) and Artificial Super Intelligence (ASI) by fostering more adaptable and generalizable AI-driven healthcare solutions [36].

In the context of arrhythmia detection, ECG-based models remain the predominant approach due to their rich morphological information and established clinical utility. However, the widespread adoption of PPG-based arrhythmia classification models is still limited, despite PPG being a more cost-effective and convenient alternative for continuous heart rhythm monitoring [37]. The lack of sufficiently optimized PPG-based machine learning models necessitates the development of novel approaches that can fully leverage the diagnostic potential of PPG signals. To address this challenge, we propose a TQCPat-based feature engineering model, which introduces a novel dynamic feature extraction methodology tailored for multi-class arrhythmia classification. Traditional feature engineering models often rely on static feature extraction techniques, limiting their adaptability and overall classification performance when compared to deep learning-based systems. To overcome this issue, our proposed TQCPat methodology incorporates dynamic lattice graph-based local feature extraction, drawing inspiration from quantum computing principles, specifically the variable superposition states of quantum particles, combined with hypergraph structures [38]. This novel approach allows for more flexible, adaptive, and computationally efficient feature extraction, making it a promising alternative to both conventional machine learning techniques and resource-intensive deep learning models.

Our model incorporates discrete wavelet transform (DWT) [39] to decompose the raw PPG signal into wavelet subbands for multilevel signal processing downstream, as well as TQCPat combined with signum, upper ternary (UT), and lower ternary (LT) functions for dynamic generation of three feature vectors for every input sample (either raw PPG signal or wavelet subband). For the three feature vectors, neighborhood component analysis (NCA) [40] and chi-squared (Chi2) [41] selector functions are applied to select six selected feature vectors, which are then fed to k-nearest neighbors (kNN) [42] and support vector machine (SVM) [43] classifiers to calculate 12 classifier-specific outcomes. The latter are input to an iterative majority voting (IMV) algorithm [44] to calculate ten additional voted outcomes, after which a greedy algorithm is applied to calculate the best model outcome from the 22 combined classifier-specific plus voted outcomes.

### Innovations and Contributions

This study introduces a novel quantum-inspired feature engineering approach, Tree Quantum Circuit Pattern (TQCPat), specifically designed for PPG-based arrhythmia detection. Unlike conventional deep learning models that rely on static feature extraction or require high computational resources, TQCPat leverages a dynamically adaptive, structured graph-based feature extraction inspired by quantum computing principles. The main contributions of our work can be summarized as follows:

- We propose a novel and quantum-inspired dynamic feature extraction function called TQCPat, which mimics quantum superposition principles and applies a structured graph-based approach for local feature extraction.
- The model has been developed using the largest public PPG signal arrhythmia dataset [30].
- Unlike traditional handcrafted feature extraction methods that rely on static transformations, TQCPat dynamically selects an optimal path in a tree-like quantum circuit, enhancing feature adaptability and improving classification accuracy.
- The combination of DWT-based multiresolution analysis and TQCPat-based dynamic feature extraction ensures that both global and local signal variations are effectively captured, which has not been explored in previous PPG-based arrhythmia detection models.
- The model automatically optimizes its feature selection process by integrating Chi-squared (Chi2) and Neighborhood Component Analysis (NCA), ensuring that only the most relevant features are retained for classification.
- Unlike conventional models that rely on a single classifier, our method employs both k-Nearest Neighbors (kNN) and Support Vector Machine (SVM) in parallel, followed by an Iterative Majority Voting (IMV) strategy to enhance classification robustness.
- Despite being a lightweight handcrafted machine learning model, our approach outperforms deep convolutional neural network (CNN)-based methods on the same dataset, achieving 91.30% accuracy while being significantly more computationally efficient.
- The TQCPat framework can be extended to other biomedical signal processing applications, including EEG-based neurological disorder detection and EMG-based muscular disorder classification, making it a versatile feature engineering tool.

In contrast to previous studies that rely on static feature extraction or computationally expensive deep learning models, our proposed approach introduces a quantum-inspired, dynamically adaptive feature engineering framework that enhances both flexibility and efficiency in PPG signal representation. Unlike traditional handcrafted machine learning methods, TQCPat leverages structured graph-based local feature extraction, mimicking quantum superposition states to capture complex signal variations more effectively. This novel approach not only surpasses conventional models in classification performance but also maintains computational efficiency, as demonstrated in our comparative analysis (see Table 6). By addressing the limitations of existing PPG-based arrhythmia classification methods, our model provides a computationally efficient, interpretable, and high-performing feature engineering solution, making it highly suitable for real-world healthcare applications, including wearable and remote monitoring systems.

## Material and Methods

### Dataset

The dataset used in this study is derived from the publicly available dataset described in Liu et al. [30], which consists of photoplethysmography (PPG) and electrocardiography (ECG) signals collected from 228 patients undergoing radiofrequency catheter ablation (RFCA) for arrhythmia treatment at Fuwai Hospital, China. A combined total of 118,217 10-s PPG and ECG waveforms were recorded. These signals were collected using a multiparameter monitoring system (BeneVision N12; Shenzhen Mindray Bio-Medical Electronics) equipped with a fingertip PPG sensor and a 5-lead ECG setup. The signals were obtained while the patients were in a supine position, and the data acquisition was conducted at a sampling rate of 250 Hz for ECG and 100 Hz for PPG. In the current study, we used only PPG waveforms, which amounted to a total of 46,827 PPG signals. The study dataset consists of six different rhythm types, which were annotated by two cardiologists based on ECG interpretations: 1: premature ventricular contraction; 2: premature atrial contraction; 3: ventricular tachycardia; 4: supraventricular tachycardia; and 5: AF (Table 1).

**Table 1:** Distribution of study PPG samples

No	Arrhythmia class	Number of signals, n	Frequency (%)
0	Sinus rhythm	14,604	31.19
1	Premature ventricular contraction	4425	9.45
2	Premature atrial contraction	3773	8.06
3	Ventricular tachycardia	2179	4.65
4	Supraventricular tachycardia	5677	12.12
5	Atrial fibrillation	16,169	34.53
Total		46,827	100

### Proposed TQCPat and TCPPat-based Feature Extraction

**Fig. 1:**
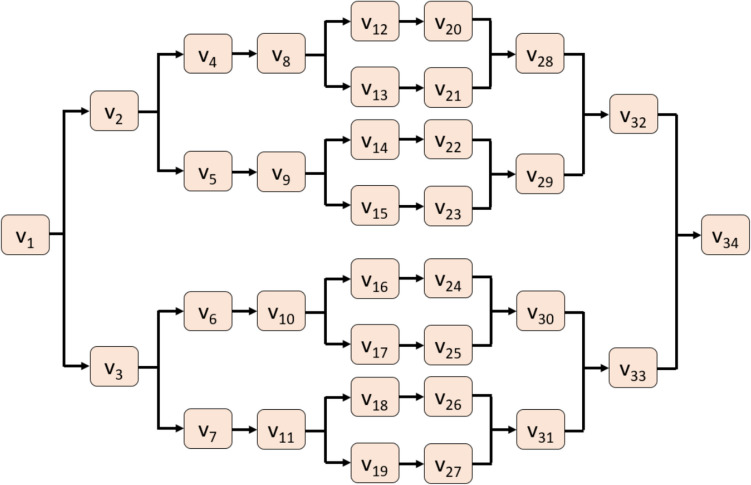
Tree quantum circuit pattern is a directed graph comprising starting (V_1_), final (V_34_) nodes and intermediary nodes (V_2–33_) squares linked by paths. The v values are sequentially populated by the 34 values of the PPG signal block

**Fig. 2:**
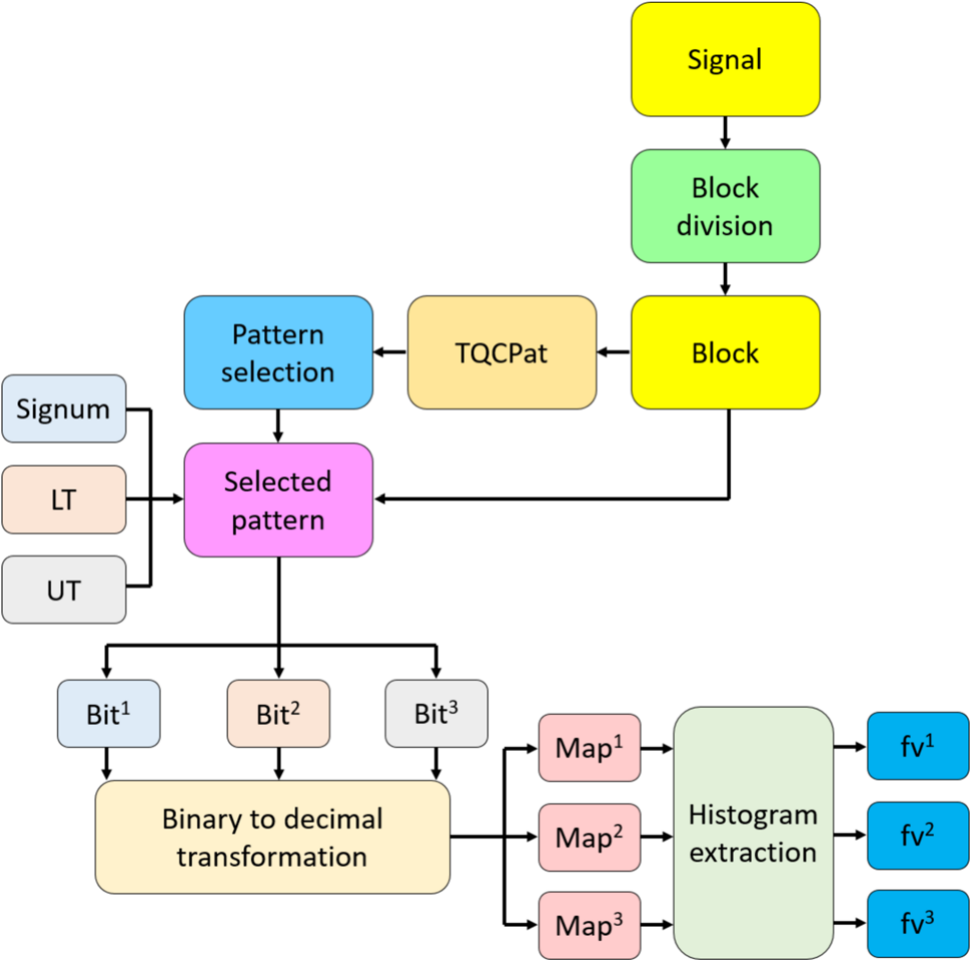
Schema overview of the proposed TQCPat-based feature extraction. **Bit, binary features; fv, feature vector. LT, lower ternary kernel; Map, feature map signals; Signum, signum kernel; UT, upper ternary kernel. The superscript numbers 1, 2, and 3 denote binary feature, feature map signal, and feature vector outputs of the signum, lower ternary, and upper ternary functions, respectively

The proposed TQCPat is a novel graph pattern modeled after a quantum circuit that features 34 nodes―multiple directed paths connect the start node to the final node via intermediary nodes―which will be populated in sequence by values in the input signal block (Fig. 1). The optimal path linking the start and final nodes is dynamically selected by applying the maximum function on these populated values, after which a local feature extractor is performed using histogram analysis (Fig. 2).

The steps of the TQCPat-based feature extraction are detailed below.

*S1:* Divide the signal into overlapping blocks, each of length 34.

1 $$v(j)=signal\left(i+j-1\right),i\in \left\{\text{1,2},\dots ,\mathcal{L}-33\right\},j\in \left\{\text{1,2},\dots ,34\right\}$$

where $v$ represents an overlapping block.

*S2:* Use the values of the block (Fig. 1) to create a tree quantum graph.

**Fig. 3:**
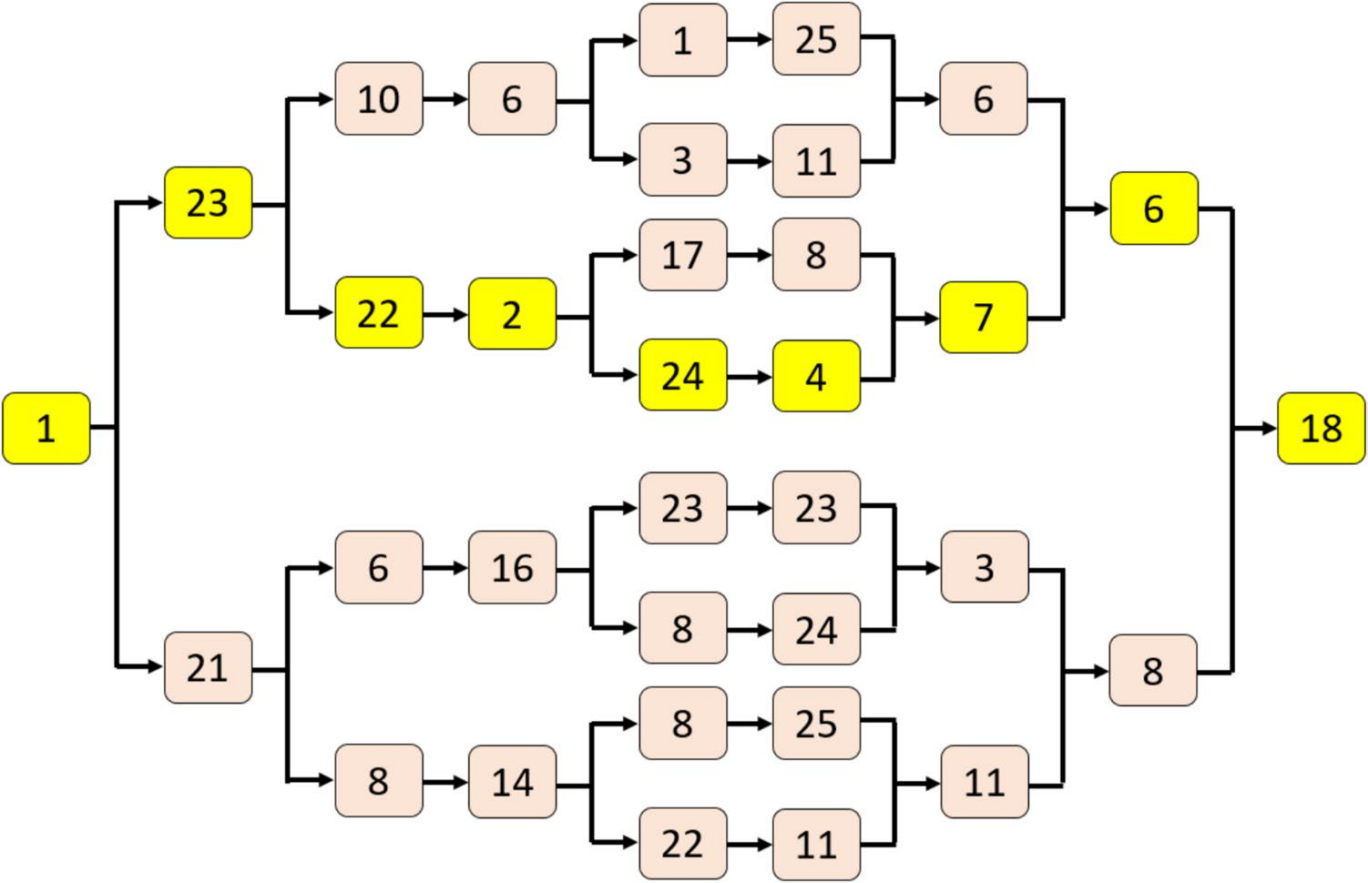
The TQCPat is populated with values of a sample signal block of length 34

*S3:* Select the optimal path using the maximum-based selection function. A numerical example is depicted in Fig. 3.

Where the paths bifurcate, the greater of two values based on the maximum function are selected to construct the optimal path linking the start and final nodes (yellow nodes). The values in the yellow nodes are used for feature extraction.

*S4:* Extract binary feature by deploying the selected values and three kernels: signum, upper ternary, and lower ternary.

2 $$\begin{array}{c}bi{t}^{k}\left(h\right)=kerne{l}_{k}\left(vs\left(h\right),vs\left(h+1\right)\right),h\in \left\{\text{1,2},\dots ,8\right\}, k\in \left\{\text{1,2},3\right\}\\ kerne{l}_{1}\left(vs\left(h\right),vs\left(h+1\right)\right)=\left\{\begin{array}{c}0,vs\left(h\right)-vs\left(h+1\right)<0\\ 1,vs\left(h\right)-vs\left(h+1\right)\ge 0\end{array}\right.\\ \begin{array}{c}kerne{l}_{2}\left(vs\left(h\right),vs\left(h+1\right)\right)=\left\{\begin{array}{c}0,vs\left(h\right)-vs\left(h+1\right)\ge -t\\ 1,vs\left(h\right)-vs\left(h+1\right)<-t\end{array}\right.\\ kerne{l}_{3}\left(vs\left(h\right),vs\left(h+1\right)\right)=\left\{\begin{array}{c}0,vs\left(h\right)-vs\left(h+1\right)\le t\\ 1,vs\left(h\right)-vs\left(h+1\right)>t\end{array}\right.\\ t=\frac{std\left(signal\right)}{2}\end{array}\end{array}$$

where $bit$ represents binary features, each of length 8; $kerne{l}_{1}(.)$; signum function; $kerne{l}_{2}(.)$, lower ternary function; $kerne{l}_{3}(.)$, upper ternary function; $std(.)$, standard deviation calculation function; and $t$, threshold value. In this step, three-bit groups are extracted.

Step 5: Calculate three map values using binary to decimal transformation.

3 $${fmap}^{k}\left(i\right)=\sum_{u=1}^{8}bi{t}^{k}(u)\bullet {2}^{8-u}$$

where $fmap$ represents the feature map signal.

*S5:* Repeat steps 1–5 until all overlapping blocks have been processed to generate three map signals per block.

*S6:* Extract the created map signals to create three feature vectors. Each feature vector is of length 256 as the corresponding feature map signal has been coded with 8 bits.

4 $$f{v}^{k}=\lambda \left(fma{p}^{k}\right)$$

where $fv$ represents feature vector; and $\lambda (.)$, histogram extraction function.

### Proposed Feature Engineering Model

**Fig. 4:**
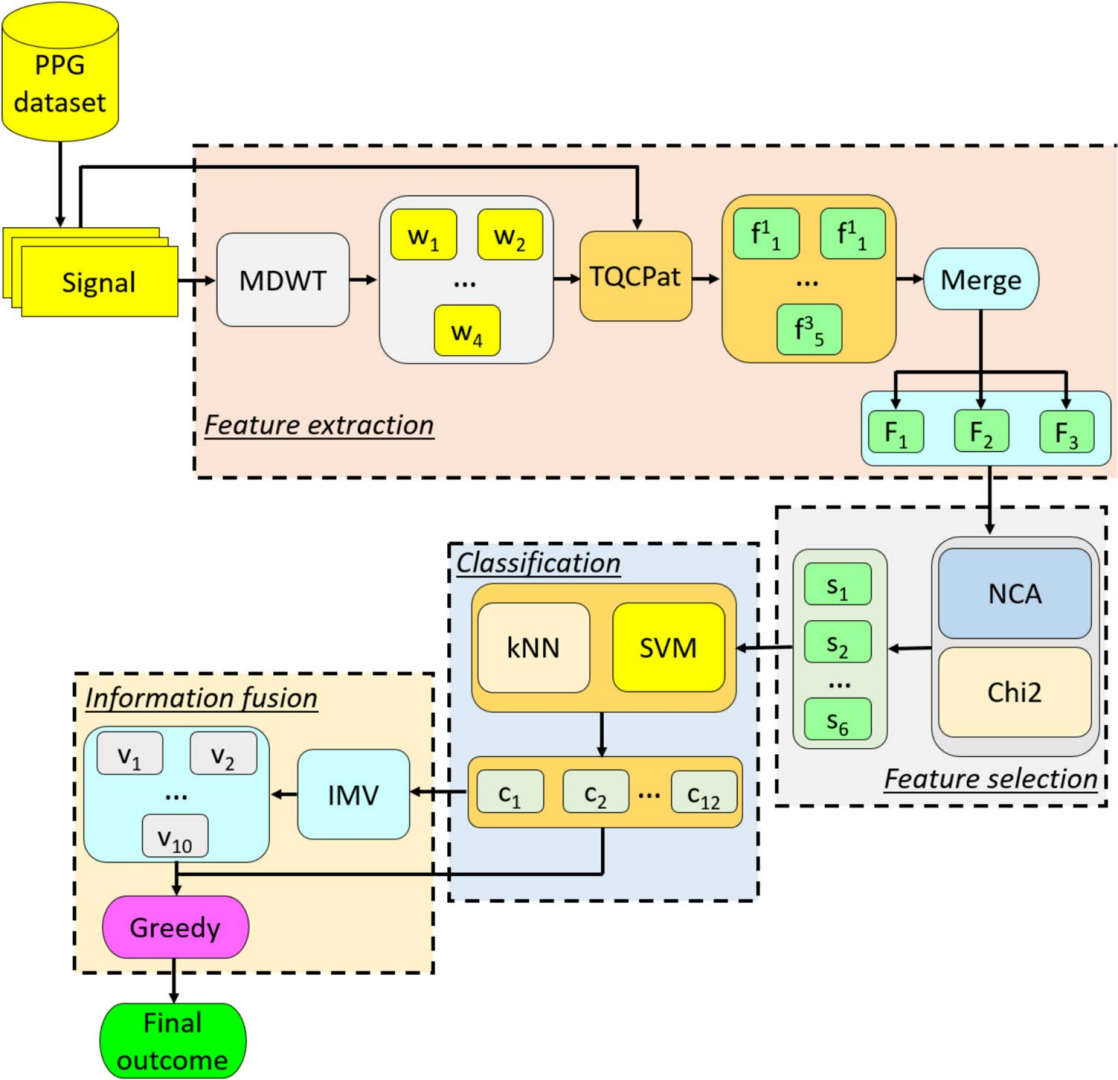
Schema of the proposed TQCPat-based feature engineering model. **c, classifier-specific outcome; f, individual feature vector; F, merged feature vector; MDWT, multilevel discrete wavelet transform; s, selected feature vector; v, voted outcomes; w, wavelet subband

The proposed system consists of four main stages: (i) multilevel feature extraction, (ii) feature selection, (iii) classification, and (iv) information fusion. In the multilevel feature extraction stage, discrete wavelet transform (DWT) is applied to decompose the PPG signals into four levels using Daubechies 4 (db4) wavelet. Additionally, the proposed Tree Quantum Circuit Pattern (TQCPat) extracts three distinct feature vectors per signal by employing Signum, Upper Ternary (UT), and Lower Ternary (LT) transformations (see Sect. "Proposed TQCPat and TCPPat-based feature extraction"). In the feature selection phase, Chi-squared (Chi2) [41] and Neighborhood Component Analysis (NCA) [40] methods are used to rank the most relevant features and reduce dimensionality, ensuring improved classifier performance. The classification stage involves training k-Nearest Neighbors (kNN) [42] and Support Vector Machine (SVM) [43] models with tenfold cross-validation to obtain 12 classifier-specific predictions. Finally, the information fusion phase [45] integrates classification results using an Iterative Majority Voting (IMV) [44] approach, where multiple classifier-specific outputs are combined, and the most frequently predicted class label is selected as the final output. A block diagram summarizing all these phases is given in Fig. 4.

The steps of the model are detailed below.

#### Feature Extraction

*Step 1:* Apply MDWT with 4 levels to the PPG signal using used the Daubechies 4 (db4) wavelet filter.

5 $$\begin{array}{c}\left[lo{w}^{1}, hig{h}^{1}\right]=\delta \left(signal\right)\\ \left[lo{w}^{g+1}, hig{h}^{g+1}\right]=\delta \left(lo{w}^{g}\right), g\in \{\text{1,2},3\}\end{array}$$

where $\delta (.)$ represents the discrete wavelet transform function; $low$, low pass filter; and $high$, high pass filter.

*Step 2:* Generate the individual feature vectors by applying the TQCPat-based feature extraction function (see Sect. "Proposed TQCPat and TCPPat-based feature extraction") to both the raw PPG signal and its corresponding MDWT-generated wavelet subbands.

6 $$\begin{array}{c}\left[{f}_{1}^{1} {f}_{1}^{2} {f}_{1}^{3}\right]=\psi \left(signal\right)\\ \left[{f}_{l+1}^{1} {f}_{l+1}^{2} {f}_{l+1}^{3}\right]=\psi \left(lo{w}_{l}\right), l\in \{\text{1,2},\text{3,4}\}\end{array}$$

where $f$ represents the generated feature vector of length 256; and $\psi (.)$,TQCPat feature extraction function.

*Step 3:* Apply categorical merging to generate three merged feature vectors.

7 $$\begin{array}{c}{F}^{g}\left(r+x\bullet \left(256\right)\right)={f}_{x}^{g}\left(r\right), r\in \left\{\text{1,2},\dots ,256\right\},\\ g\in \left\{\text{1,2},3\right\}, x \in \left\{\text{1,2},\dots ,5\right\}\end{array}$$

where $F$ represents merged feature vector of length 1280 (= 256 × 5).

*Step 4:* Repeat Steps 1–3 until all PPG signals have been processed to generate three feature matrices ($X$).

#### Feature Selection

*Step 5:* Calculate six qualified indices of the three extracted feature matrices using NCA [40] and Chi2 [41] feature selectors.

8 $$\begin{array}{c}id{x}_{2g-1}=Chi2({X}_{g},y)\\ id{x}_{2g}=NCA({X}_{g},y)\end{array}$$

where $idx$ represents the qualified indices of the generated feature matrices; and $y,$ actual output.

*Step 6:* Select the top 256 features from each feature matrix using the corresponding qualified index.

9 $$\begin{array}{c}{s}_{2g-1}\left(d,z\right)={X}_{g}\left(d,id{x}_{2g-1}\left(z\right)\right), d\in \left\{\text{1,2},\dots ,N\right\}, z\in \left\{\text{1,2},\dots ,256\right\}\\ {s}_{2g}\left(d,z\right)={X}_{g}\left(d,id{x}_{2g}\left(z\right)\right)\end{array}$$

where $s$ represents the selected feature vector (six feature vectors are selected, each of length 256); and $N$, number of the used PPG signal.

#### Classification

kNN [42] and SVM [43] are applied to the six selected feature vectors, generating 12 (6 × 2) outcomes.

*Step 7:* Calculate 12 classifier-specific outcomes from the six selected feature vectors using kNN and SVM classifiers with ten-fold cross-validation (CV).

10 $$\begin{array}{c}{c}_{r}=kNN\left({s}_{r},y\right), r\in \{\text{1,2},\dots ,6\}\\ {c}_{r+6}=SVM({s}_{r},y)\end{array}$$

where $c$ represents classifier-specific outcomes.

#### Information Fusion

IMV [44] is applied to generate 10 voted outcomes from the 12 classifier-specific outcomes; and greedy algorithm, to select the best outcome from the total 22 (12 classifier-specific plus 10 voted outcomes). The steps are detailed below:

*Step 8:* Apply the IMV algorithm to generate 10 voted results.

11 $$\begin{array}{c}acc\left(b\right)=\theta \left({c}_{b},y\right),b\in \{\text{1,2},\dots ,12\}\\ id=\rho \left(acc\right)\\ {v}^{a-2}=\varpi \left({c}_{id\left(1\right)},{c}_{id\left(2\right)},\dots ,{c}_{id\left(a\right)}\right), a\in \{\text{3,4},\dots ,12\}\end{array}$$

where $acc$ represents classification accuracy; $\theta \left(.\right),$ classification accuracy calculation function; $id$: the qualified indices; $\rho (.)$, sorting by descending function; $v$, voted outcome; and $\varpi (.)$: mode function.

*Step 9:* Select the best outcome using the greedy algorithm.

12 $$\begin{array}{c}acc\left(z+12\right)=\theta \left({v}_{z},y\right),z\in \{\text{1,2},\dots ,10\}\\ ind=max\left(acc\right)\\ inres=\left\{\begin{array}{c}{c}_{ind}, ind\le 12\\ {v}_{ind-12},ind>12\end{array}\right.\end{array}$$

where $ind$ represents the index of the maximum accuracy; and $finres$, the final outcome.

## Experimental Results

The model was implemented in MATLAB (2023b). The parameters used to develop the model are listed in Table 2.

**Table 2:** Parameters used to develop the proposed TQCPat-based model

Phase	Method	Parameters
**Feature extraction**	MDWT	Filter: db4, Number of levels: 4
	TQCPat	Length of the overlapping block: 34, Graph generation function: maximum, Kernel: Signum, lower ternary, upper ternary Feature vectors: three feature vectors are extracted, each of length 256
	Feature merging	Three feature vectors have been generated, each of length 1280 (256 × 5)
**Feature selection**	NCA	Three selected feature vectors are created, each of length 256
	Chi2	Three selected feature vectors are created, each of length 256
**Classification**	kNN	k: 10, distance: L1-norm: Manhattan, voting: squared inverse, validation: tenfold cross-validation
	SVM	Kernel: second-degree polynomial, kernel scale: automatic, box constraint: 1, coding: one-vs-one, Validation: tenfold CV
**Information fusion**	IMV	Iteration: from 3 to 12, qualification: classification accuracy-based sorting, voting function: mode function
	Greedy algorithm	Choose the most accurate final outcome

**Fig. 5:**
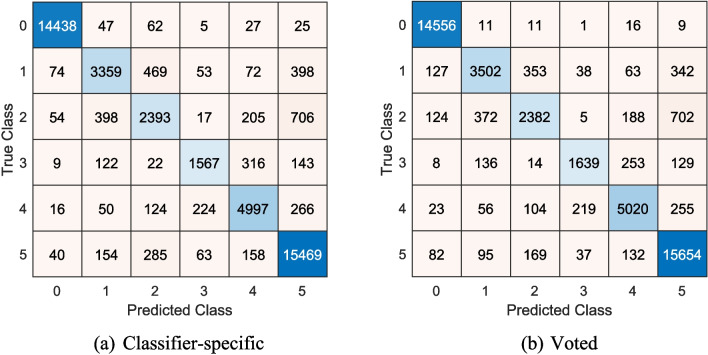
Confusion matrices for the best results calculated from the classifier-specific (a) and voted outcomes (b)

The model attained excellent classification performance in terms of accuracy and F1-score (Table 3), with the best-voted outcome results―classification accuracy and overall F1-score of 91.30% and 85.55%, respectively ―surpassing that of the classifier-specific outcomes. Similarly, the confusion matrices of the classifier-specific and voted outcomes suggest that the latter has few instances of misclassification (Fig. 5). These results lend support to our design decision to incorporate IMV into our model.

**Table 3:** Overall results stratified by classifier-specific and voted outcomes. The top results are in bold fonts

Classifier-specific outcomes			Voted outcomes		
No	**Accuracy (%)**	**F1-score (%)**	**No**	**Accuracy (%)**	**F1-score (%)**
1	86.35	79.01	1	90.72	84.61
2	88.46	81.79	2	91.13	85.19
3	87.59	80.32	3	91.26	85.26
4	88.26	81.21	4	91.27	85.43
5	86.48	79.40	5	**91.30**	85.46
6	87.41	80.53	6	**91.30**	**85.55**
7	88.38	81.47	7	91.09	85.32
8	**90.17**	**83.63**	8	91.04	85.39
9	89.74	82.88	9	90.78	85.08
10	89.59	82.71	10	90.49	84.70
11	89.85	83.51	-	-	-
12	89.79	83.32	-	-	-

## Discussion

**Fig. 6:**
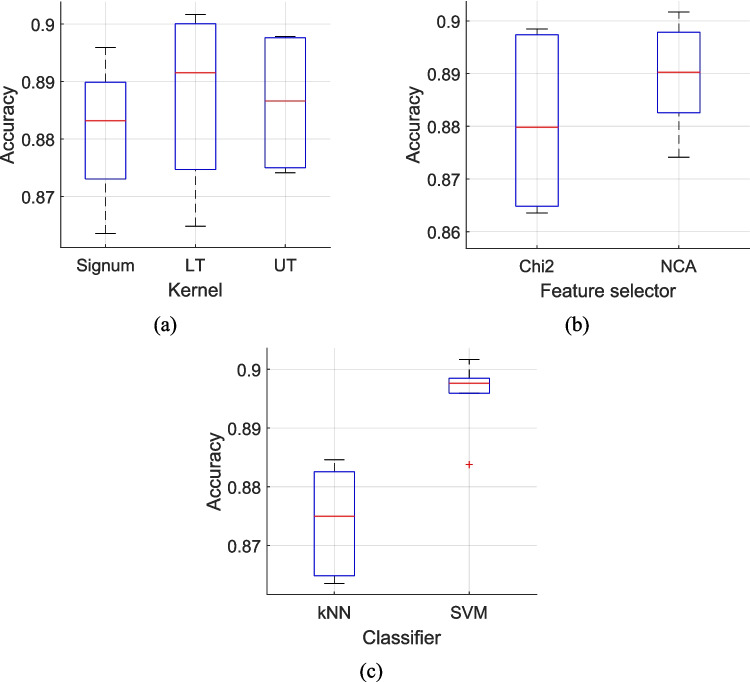
Mean accuracy results of classifier-specific outcomes stratified by deployed feature extraction function (**a**), feature selection function (**b**), and (**c**) classifier (**c**). LT, lower ternary function; Signum, signum function; UT, upper ternary function

This work introduced a novel quantum-inspired dynamic TQCPat-based feature extraction function, which we used to construct a handcrafted machine learning model that calculates 22 (12 classifier-specific plus 10 voted), and selects the most accurate one in a self-organized manner. By incorporating upstream MDWT-based signal processing, the model is able to generate multiple high- and low-level features, mimicking the deep learning model, which has contributed to the high classification performance. To generate the 12 classifier-specific outcomes, the feature extraction kernels, feature selectors and classifiers are combined (Table 4). Overall, lower ternary, NCA, and SVM functions are the kernel, feature selector, and classifier, respectively, that yielded the highest mean accuracies (Fig. 6).

**Table 4:** Combinations of functions used to generate the classifier-specific outcomes

No	Feature extraction kernel	Feature selector	Classifier
1	Signum	Chi2	kNN
2	Lower ternary	NCA	kNN
3	Upper ternary	Chi2	kNN
4	Signum	NCA	kNN
5	Lower ternary	Chi2	kNN
6	Upper ternary	NCA	kNN
7	Signum	Chi2	SVM
8	Lower ternary	NCA	SVM
9	Upper ternary	Chi2	SVM
10	Signum	NCA	SVM
11	Lower ternary	Chi2	SVM
12	Upper ternary	NCA	SVM

The results obtained based on Table 4 are given in Fig. 6.

The results shown in Fig. 6 were obtained by averaging accuracies across all possible combinations for each component:

- For feature extraction kernel comparison (Signum, LT, UT), the accuracy was averaged across all combinations of feature selectors (Chi2, NCA) and classifiers (kNN, SVM). For example, Signum's performance represents the mean accuracy of four combinations: Signum + Chi2 + kNN, Signum + NCA + kNN, Signum + Chi2 + SVM, and Signum + NCA + SVM.
- For feature selector comparison (Chi2, NCA), results were averaged across all combinations of feature extraction kernels (Signum, LT, UT) and classifiers (kNN, SVM). Each feature selector's performance represents the mean of six combinations (3 kernels × 2 classifiers).
- For classifier comparison (kNN, SVM), accuracies were averaged across all combinations of feature extraction kernels (Signum, LT, UT) and feature selectors (Chi2, NCA). Each classifier's performance represents the mean of six combinations (3 kernels × 2 feature selectors).

This comprehensive averaging approach ensures an unbiased comparison of each component's performance while accounting for all possible configurations presented in Table 4. The best outcome is the majority voted outcome is the sixth voted outcome, which was calculated from the top 8 (= 6 + 3–1) classifier-specific outcomes (Table 5). Among the most frequently deployed kernels are signum along with lower ternary; feature selector, NCA; and classifier, SVM (Fig. 6), which reflect the mean accuracies results of these individual functions (Fig. 6).

**Table 5:** Classifier-specific outcomes (in descending order of accuracy) used to generate the final voted outcome

Classifier-specific outcome	Feature extractor	Feature selector	Classifier
8	Lower ternary	NCA	SVM
11	Lower ternary	Chi2	SVM
12	Upper ternary	NCA	SVM
9	Upper ternary	Chi2	SVM
10	Signum	NCA	SVM
2	Lower ternary	NCA	kNN
7	Signum	Chi2	SVM
4	Signum	NCA	kNN

To highlight the effectiveness of our proposed model, we compared its performance with previous studies that utilized PPG signals for arrhythmia detection. Table 6 provides a detailed comparison of our model with existing methods in the literature.

**Table 6:** Comparison of state-of-the-art methods on the same dataset

Study	Dataset	Method	Validation	Result(s)	Computational Cost
Liu et al. [30]	• 6 rhythm types • PPG and ECG waveforms	Signal preprocessing, Deep convolutional neural network (Custom designed)	Hold-out CV (60:20:20)	Acc. = 85.0 Sen. = 75.8 Spe. = 96.9	High (Deep Model)
Wu et al. [25]	• 5 rhythm types • PPG waveforms	A hybrid deep learning model (Res-BiANet)	Hold-out CV (60:20:20)	Acc. = 92.38 Pre. = 88.46 Sen. = 85.15 Spe. = 98.43 F1S. = 86.88	High (Deep Model)
Liu et al. [46]	• 6 rhythm types • PPG and ECG waveforms	Hybrid convolutional neural network (CNN)-transformer network	Hold-out CV (60:20:20)	Pre. = 87.0 Rec. = 87.1 F1S. = 86.8	High (Deep Model)
Our method	• 6 rhythm types • PPG waveforms	MDWT based TQCPat, Chi2, NCA, kNN, SVM, IMV	tenfold CV	Acc. = 91.30 F1S. = 85.55	Low (Handcrafted ML)

*Acc: Accuracy, Sen: Sensitivity, Spe: Specificity, Pre: Precision, Rec: Recall, F1S: F1-Score

Finally, our model’s multiclass arrhythmia classification accuracy of 91.30% is higher than the 85% attained by the convolutional neural network-based deep model by Liu et al. [30], which had also been trained on the same PPG database. In addition, the method proposed in [30] had high computational complexity. Wu et al. [25] achieved higher classification performance than us. However, the model developed in [25] classified 5 rhythm types and had high computational complexity. Liu et al. [46] used PPG and ECG waveforms together in their study and provided a value (86.8%) very close to our F1 Score (85.55%). Our study uses only the PPG waveform. Our model’s results provide support for using well-designed and computationally less demanding handcrafted adaptive and dynamic feature engineering machine learning models as an alternative to deep models for arrhythmia detection.

Our dataset exhibits class imbalance, particularly in the distribution of different arrhythmia types (see Table 1). However, rather than artificially balancing the dataset through oversampling or weighting techniques, we opted to evaluate the model’s inherent robustness in handling imbalanced data. The rationale behind this decision is twofold: (1) real-world arrhythmia prevalence is naturally imbalanced, and a model that successfully classifies minority classes without artificially altering data distributions is more clinically applicable, and (2) oversampling can introduce overfitting risks, leading to models that fail to generalize effectively. To ensure that the model is robust against class imbalance, we employed tenfold CV, allowing the model to learn from different distributions while preventing overfitting. Additionally, our evaluation prioritizes the F1-score, which provides a balanced metric that accounts for both precision and recall. The consistently high F1-scores across all rhythm classes demonstrate that our model successfully identifies minority classes without requiring explicit class balancing techniques (see Table 3). Furthermore, our feature engineering approach plays a crucial role in mitigating class imbalance effects. Unlike deep learning models that often rely on high-volume data, our quantum-inspired feature selection framework enhances the separability of arrhythmia types, leading to improved performance even in minority classes. The confusion matrix (Fig. 5) further supports this, showing that the model achieves high sensitivity and specificity across all rhythm types, including those with lower representation in the dataset. Overall, the results indicate that our model effectively handles class imbalance through inherent generalization, robust feature extraction, and comprehensive cross-validation strategies, without the need for explicit resampling methods. Beyond its ability to effectively handle class imbalance, our model also demonstrates computational efficiency, which is a crucial factor for real-world deployment. In this context, the computational complexity of the proposed TQCPat-based feature engineering model is primarily influenced by three key phases: (i) feature extraction, (ii) feature selection, and (iii) classification and decision fusion. Below is a breakdown of the computational costs associated with each stage.

### Feature Extraction Complexity

The proposed TQCPat-based feature extraction method operates on overlapping blocks of 34 samples from each PPG signal. The MDWT is first applied to decompose the raw signal into multiple subbands, each of which undergoes dynamic feature extraction using graph-based path selection. The TQCPat function performs three key operations:

- Graph population: $O(n)$, where $n$ is the number of samples per block.
- Optimal path selection (maximum function): $O(n)$
- Feature vector generation using histogram-based extraction: $O(nlogn)$

Given that MDWT decomposes the signal into four levels, the overall complexity of the feature extraction phase can be approximated as:

$$O\left(nlogn\right)+O\left(n\right)+O\left(n\right)=O(nlogn)$$

### Feature Selection Complexity

The model employs Chi2 and NCA for selecting the most relevant features. The complexities of these techniques are:

- Chi2 selection: $O(nk)$, where $k$ is the number of classes.
- NCA selection: $$O({n}^{2})$$

Since Chi2 operates in linear time and NCA is computationally more expensive, the dominant factor in feature selection is NCA, yielding an approximate complexity of:

$$O\left(nk\right)+O\left({n}^{2}\right)=O({n}^{2})$$

### Classification and Decision Fusion Complexity

The classification phase involves training k-NN and SVM models and decision fusion:

- kNN classification: $O(nd)$, where $d$ is the number of features.
- SVM classification: $O({n}^{3})$ for training phase.
- IMV and Greedy algorithm: $O(n)$

Since the SVM training phase is $O({n}^{3})$, the total computational complexity:

$$O\left(nd\right)+O\left({n}^{3}\right)+O\left(n\right)=O({n}^{3})$$

### Overall Complexity Analysis

Combining all stages, the dominant computational cost stems from:

- Feature extraction: $$O(nlogn)$$
- Feature selection: $$O({n}^{2})$$
- Classification and decision: $O({n}^{3})$

As a result, overall computational complexity:

$$O\left(nlogn\right)+O\left({n}^{2}\right)+O\left({n}^{3}\right)=O({n}^{3})$$

A CNN (Deep Neural Network) or Transformer-based model is usually $O({n}^{5})$ or has a higher computational cost. Therefore, the proposed model is lighter, requires less computational power and runs faster than deep learning models. Thus, the TQCPat-based model offers a more computationally efficient alternative.

The findings, advantages, limitations and future works of the developed model are listed below.

### Findings

- Proposed TQCPat-based model achieved a classification accuracy of 91.30% and an F1-score of 85.55%.
- Presented TQCPat is an effective dynamic feature extraction function for processing PPG signals.
- The developed model outperformed a state-of-the-art deep model [30], in terms of classification accuracy.
- Employed elements such as lower ternary, NCA and SVM are the best feature extraction kernel, feature selector, and classifier, respectively.

### Advantages

- Quantum-inspired TQCPat is a novel accurate feature extraction function.
- Ability of the model to generate multiple outcomes and select the most accurate one automatically enhances its efficiency and reliability.
- The model performed well for classifying a huge PPG dataset for six arrhythmia classes.

### Limitations

- The computational complexity of the developed system is linearly increased with the incorporation of multiple machine learning steps. Nevertheless, this model has lower time complexity than deep learning models.

### Future Works

The proposed TQCPat-based model demonstrates promising potential in various biomedical applications beyond arrhythmia classification using PPG signals. In future studies, we plan to explore additional real-world applications of our method, including:

- The proposed model can be integrated into wearable health monitoring devices, such as smartwatches and fitness trackers, for continuous arrhythmia screening and real-time heart health monitoring.
- Since the method is computationally efficient, it can be deployed on low-power embedded systems to provide on-device arrhythmia detection without requiring cloud-based processing.
- The TQCPat framework can be extended to electroencephalogram (EEG) signal analysis to detect epilepsy, Alzheimer’s disease, and sleep disorders.
- By modifying the feature extraction approach, our method can help in developing lightweight AI models for real-time neurological diagnostics.
- Our approach can be applied to electromyographic (EMG) signals to assist in diagnosing neuromuscular diseases such as ALS and muscular dystrophy.
- The efficient feature extraction and classification mechanism make it suitable for early-stage detection and monitoring of muscular disorders.
- The integration of our method into telemedicine platforms can allow physicians to remotely monitor patients with cardiovascular risks.
- The ability to analyze large-scale PPG data efficiently makes it a valuable tool for automated arrhythmia screening in clinical and home-care settings.
- The proposed model can be implemented in Intensive Care Units (ICU) monitoring systems for early detection of critical cardiac abnormalities using PPG signals.
- The fast computational performance enables near real-time processing of patient vitals in emergency care units.

## Conclusions

This work presents a novel quantum-inspired TQCPat-based model for the automated detection of arrhythmia classes using PPG signals. Our developed model attained a classification accuracy of 91.30% and an F1-score of 85.55% in classifying six classes using 46,827 PPG signals (228 patients).

The limitation of the model is obtaining high performance for all classes and also in handling imbalanced datasets. Hence, we plan to refine the proposed model and use a larger and more diverse PPG dataset in the future for accurate arrhythmia detection.

## Data Availability

No datasets were generated or analysed during the current study.

## References

[1] S. R. Sankranti *et al.*, "Effective IoT Based Analysis of Photoplethysmography Waveforms for Investigating Arterial Stiffness and Pulse Rate Variability," *SN Computer Science,* vol. 5, no. 5, p. 474, 2024.

[2] A. Goshvarpour and A. Goshvarpour, "Asymmetric measures of polar Chebyshev chaotic map for discrete/dimensional emotion recognition using PPG," *Biomedical Signal Processing and Control,* vol. 100, p. 107089, 2025.

[3] M. Khan, B. K. Singh, and N. Nirala, "Empirical wavelet decomposition of photoplethysmographic signal for hypertension risk stratification and detection of diabetes mellitus using machine learning techniques," *International Journal of Medical Engineering and Informatics,* vol. 17, no. 1, pp. 74-88, 2025.

[4] A. S. Machikhin et al., "Combined Optical and Acoustic Microscopy for Non-Invasive Cardiovascular Studies Using Zebrafish Model," IEEE Transactions on Instrumentation and Measurement, 2024.

[5] G. Lu, F. Yang, J. A. Taylor, and J. F. Stein, "A comparison of photoplethysmography and ECG recording to analyse heart rate variability in healthy subjects," *Journal of medical engineering & technology,* vol. 33, no. 8, pp. 634-641, 2009. 19848857 10.3109/03091900903150998

[6] M. Mohammadpoor Faskhodi *et al.*, "On the use of fractional calculus to improve the pulse arrival time (PAT) detection when using photoplethysmography (PPG) and electrocardiography (ECG) signals," *Plos one,* vol. 19, no. 2, p. e0298354, 2024. 10.1371/journal.pone.0298354 PMC10871495 38363753

[7] S. N. Kounalakis and N. D. Geladas, "The role of pulse transit time as an index of arterial stiffness during exercise," *Cardiovascular Engineering,* vol. 9, pp. 92-97, 2009. 19657732 10.1007/s10558-009-9081-4

[8] T. Pereira *et al.*, "Photoplethysmography based atrial fibrillation detection: a review," *NPJ digital medicine,* vol. 3, no. 1, pp. 1-12, 2020. 31934647 10.1038/s41746-019-0207-9 PMC6954115

[9] N. L. Kazanskiy, S. N. Khonina, and M. A. Butt, "A review on flexible wearables-Recent developments in non-invasive continuous health monitoring," Sensors and Actuators A: Physical, p. 114993, 2024.

[10] Y. Zhang et al., "Personalized Continuous Blood Pressure Tracking through Single channel PPG in Wearable Scenarios," IEEE Journal of Biomedical and Health Informatics, 2025. 10.1109/JBHI.2025.3535788 40031338

[11] R. Manoj, K. V. Raj, P. Nabeel, M. Sivaprakasam, and J. Joseph, "Measurement of pressure dependent variations in local pulse wave velocity within a cardiac cycle from forward travelling pulse waves," *Scientific Reports,* vol. 15, no. 1, p. 3066, 2025. 39856220 10.1038/s41598-025-87143-z PMC11759701

[12] M. Hammad, A. Maher, K. Wang, F. Jiang, and M. Amrani, "Detection of abnormal heart conditions based on characteristics of ECG signals," *Measurement,* vol. 125, pp. 634-644, 2018.

[13] Y. Li, Y. Li, X. He, J. Fang, C. Zhou, and C. Liu, "Learner’s cognitive state recognition based on multimodal physiological signal fusion," *Applied Intelligence,* vol. 55, no. 2, pp. 1-16, 2025.

[14] M. Kaisti *et al.*, "Clinical assessment of a non-invasive wearable MEMS pressure sensor array for monitoring of arterial pulse waveform, heart rate and detection of atrial fibrillation," *NPJ digital medicine,* vol. 2, no. 1, p. 39, 2019. 31304385 10.1038/s41746-019-0117-x PMC6550190

[15] R. K. Pandey and P. C.-P. Chao, "External temperature sensor assisted a new low power photoplethysmography readout system for accurate measurement of the bio-signs," *Microsystem Technologies,* vol. 27, pp. 2315-2343, 2021. 33281302 10.1007/s00542-020-05106-y PMC7695241

[16] P. Ebrahimi *et al.*, "Plasma exchange as a rescue therapy for treatment-resistant thyroid storm with concurrent heart failure: a literature review based on a case report," *International Journal of Emergency Medicine,* vol. 17, no. 1, p. 195, 2024. 39710667 10.1186/s12245-024-00783-2 PMC11664921

[17] S. Sahoo, M. Dash, S. Behera, and S. Sabut, "Machine learning approach to detect cardiac arrhythmias in ECG signals: A survey," *Irbm,* vol. 41, no. 4, pp. 185-194, 2020.

[18] V. Gupta, "Wavelet transform and vector machines as emerging tools for computational medicine," *Journal of Ambient Intelligence and Humanized Computing,* vol. 14, no. 4, pp. 4595-4605, 2023.

[19] V. Kittipibul and C. S. Lam, "Heart failure with preserved ejection fraction and atrial fibrillation: epidemiology, pathophysiology, and diagnosis interplay," Heart Failure Reviews, pp. 1–9, 2025. 10.1007/s10741-025-10488-0 39849281

[20] N. Ahmed and Y. Zhu, "Early detection of atrial fibrillation based on ECG signals," *Bioengineering,* vol. 7, no. 1, p. 16, 2020. 32069949 10.3390/bioengineering7010016 PMC7148541

[21] M. H. Ilyas *et al.*, "Screening for atrial fibrillation: risks, benefits, and implications on future clinical practice," *Current Treatment Options in Cardiovascular Medicine,* vol. 26, no. 8, pp. 233-242, 2024.

[22] M. A. Serhani, H. T. El Kassabi, H. Ismail, and A. Nujum Navaz, "ECG monitoring systems: Review, architecture, processes, and key challenges," *Sensors,* vol. 20, no. 6, p. 1796, 2020. 32213969 10.3390/s20061796 PMC7147367

[23] R. Kher, "Signal processing techniques for removing noise from ECG signals," *J. Biomed. Eng. Res,* vol. 3, no. 101, pp. 1-9, 2019.

[24] D. Seok, S. Lee, M. Kim, J. Cho, and C. Kim, "Motion artifact removal techniques for wearable EEG and PPG sensor systems," *Frontiers in Electronics,* vol. 2, p. 685513, 2021.

[25] Y. Wu, Q. Tang, W. Zhan, S. Li, and Z. Chen, "Res-BiANet: A Hybrid Deep Learning Model for Arrhythmia Detection Based on PPG Signal," *Electronics,* vol. 13, no. 3, p. 665, 2024.

[26] R. Kanawade, S. Tewary, and H. K. Sardana, "Photoplethysmography based arrhythmia detection and classification," 2019: IEEE, pp. 944–948.

[27] N. Paradkar and S. R. Chowdhury, "Cardiac arrhythmia detection using photoplethysmography," in 2017 39th Annual International Conference of the IEEE Engineering in Medicine and Biology Society (EMBC), 2017: IEEE, pp. 113–116. 10.1109/EMBC.2017.8036775 29059823

[28] Neha, H. K. Sardana, N. Dogra, and R. Kanawade, "Dynamic time warping based arrhythmia detection using photoplethysmography signals," Signal, Image and Video Processing, vol. 16, no. 7, pp. 1925–1933, 2022.

[29] D. Han et al., "Smartwatch PPG peak detection method for sinus rhythm and cardiac arrhythmia," in 2019 41st Annual International Conference of the IEEE Engineering in Medicine and Biology Society (EMBC), 2019: IEEE, pp. 4310–4313. 10.1109/EMBC.2019.8857325 31946821

[30] Z. Liu *et al.*, "Multiclass arrhythmia detection and classification from photoplethysmography signals using a deep convolutional neural network," *Journal of the American Heart Association,* vol. 11, no. 7, p. e023555, 2022. 35322685 10.1161/JAHA.121.023555 PMC9075456

[31] S. Asif et al., "Advancements and prospects of machine learning in medical diagnostics: unveiling the future of diagnostic precision," Archives of Computational Methods in Engineering, pp. 1–31, 2024.

[32] K. Ouanes and N. Farhah, "Effectiveness of Artificial Intelligence (AI) in clinical decision support systems and care delivery," *Journal of Medical Systems,* vol. 48, no. 1, p. 74, 2024. 39133332 10.1007/s10916-024-02098-4

[33] S. S. Bhuyan *et al.*, "Generative Artificial Intelligence Use in Healthcare: Opportunities for Clinical Excellence and Administrative Efficiency," *Journal of Medical Systems,* vol. 49, no. 1, p. 10, 2025. 39820845 10.1007/s10916-024-02136-1 PMC11739231

[34] S. Takahashi *et al.*, "Comparison of vision transformers and convolutional neural networks in medical image analysis: a systematic review," *Journal of Medical Systems,* vol. 48, no. 1, p. 84, 2024. 39264388 10.1007/s10916-024-02105-8 PMC11393140

[35] J. C. Delmoral and J. M. RS Tavares, "Semantic Segmentation of CT Liver Structures: A Systematic Review of Recent Trends and Bibliometric Analysis: Neural Network-based Methods for Liver Semantic Segmentation," *Journal of Medical Systems,* vol. 48, no. 1, p. 97, 2024. 39400739 10.1007/s10916-024-02115-6 PMC11473507

[36] X. Li et al., "Artificial general intelligence for medical imaging analysis," IEEE Reviews in Biomedical Engineering, 2024. 10.1109/RBME.2024.3493775 39509310

[37] M. Khalili *et al.*, "Detecting cardiac states with wearable photoplethysmograms and implications for out-of-hospital cardiac arrest detection," *Scientific Reports,* vol. 14, no. 1, p. 23185, 2024. 39369015 10.1038/s41598-024-74117-w PMC11455951

[38] M. Rossi, M. Huber, D. Bruß, and C. Macchiavello, "Quantum hypergraph states," *New Journal of Physics,* vol. 15, no. 11, p. 113022, 2013.

[39] M. J. Shensa, "The discrete wavelet transform: wedding the a trous and Mallat algorithms," *IEEE Transactions on signal processing,* vol. 40, no. 10, pp. 2464-2482, 1992.

[40] J. Goldberger, G. E. Hinton, S. Roweis, and R. R. Salakhutdinov, "Neighbourhood components analysis," *Advances in neural information processing systems,* vol. 17, pp. 513-520, 2004.

[41] H. Liu and R. Setiono, "Chi2: Feature selection and discretization of numeric attributes," in Proceedings of 7th IEEE international conference on tools with artificial intelligence, 1995: IEEE, pp. 388–391.

[42] L. E. Peterson, "K-nearest neighbor," *Scholarpedia,* vol. 4, no. 2, p. 1883, 2009.

[43] W. S. Noble, "What is a support vector machine?," *Nature biotechnology,* vol. 24, no. 12, pp. 1565-1567, 2006. 17160063 10.1038/nbt1206-1565

[44] A. Dogan *et al.*, "PrimePatNet87: Prime pattern and tunable q-factor wavelet transform techniques for automated accurate EEG emotion recognition," *Computers in Biology and Medicine,* vol. 138, p. 104867, 2021. 34543892 10.1016/j.compbiomed.2021.104867

[45] M. Salvi et al., "Multi-modality approaches for medical support systems: A systematic review of the last decade," Information Fusion, p. 102134, 2023.

[46] Z.-D. Liu, B. Zhou, J.-K. Liu, H. Zhao, Y. Li, and F. Miao, "A CNN and Transformer Hybrid Network for Multi-Class Arrhythmia Detection from Photoplethysmography," 2024: IEEE, pp. 1–5. 10.1109/EMBC53108.2024.10782549 40031449

